# Siglec-7 orchestrates mitochondrial dynamics and metabolic reprogramming to restrain human NK cell cytotoxic function

**DOI:** 10.1016/j.jbc.2026.113543

**Published:** 2026-09-08

**Authors:** Shih-Chi Su, Nai-Ting Tsao, Tzu-Ling Wang, Yi-Chen Shih, Yuh-Ching Twu

**Affiliations:** 1 Whole-Genome Research Core Laboratory of Human Diseases, Chang Gung Memorial Hospital, Keelung, Taiwan; 2 Department of Biotechnology and Laboratory Science in Medicine, School of Biomedical Science and Engineering, National Yang Ming Chiao Tung University, Taipei, Taiwan

**Keywords:** metabolic reprogramming, mitochondrial dynamics, NK cell, Siglec-7

## Abstract

Natural killer (NK) cells are innate lymphocytes that directly eliminate tumor and virus-infected cells by integrating signals from activating and inhibitory receptors, and their effector functions are tightly coupled to cellular metabolism. Given that the inhibitory receptor PD-1 reprograms T cell metabolism to shape functional fate, the bioenergetic consequences of inhibitory receptor engagement on human NK cells remain largely unexplored, particularly for sialic acid-binding immunoglobulin-like lectin (Siglec-7), a glyco-immune checkpoint receptor. Here, we investigated metabolic programs and effector functions associated with Siglec-7 expression and antibody-mediated Siglec-7 ligation in primary NK cells and NK-92MI cells. Siglec-7^POS^ NK cells exhibited selectively impaired CD107a degranulation under glycolytic and oxidative phosphorylation inhibition, whereas Siglec-7^NEG^ cells remained relatively resistant, indicating distinct energetic wiring between these subsets. Engagement of Siglec-7 by an agonistic antibody induced mitochondrial fission with altered Drp1 phosphorylation, transient mitochondrial depolarization, and broadly suppressed mitochondrial respiration, while concurrently enhancing glycolytic capacity, consistent with a dual metabolic shift upon Siglec-7 ligation. In contrast, sustained Siglec-7 expression in NK-92MI-S cells was associated with globally enhanced mitochondrial respiratory capacity, indicating that sustained Siglec-7 expression and short-term treatment with an agonistic anti-Siglec-7 antibody were associated with distinct metabolic profiles in NK cells. Furthermore, Siglec-7^POS^ NK cells showed increased accumulation of autophagic vacuole, reduced proliferation, and heightened apoptotic susceptibility compared with Siglec-7^NEG^ counterparts. Collectively, these findings support an association between Siglec-7 status, mitochondrial homeostasis, and metabolic fitness in NK cells, with Siglec-7^NEG^ cells retaining a metabolically robust, cytotoxic phenotype.

Natural killer (NK) cells are innate lymphoid cells that mediate rapid cytotoxic responses against virus-infected and malignant cells through a diverse repertoire of germline-encoded activating and inhibiting receptors (1, 2). NK effector functions are tightly coupled to cellular metabolism. Conventional NK cells largely rely on glycolysis to support degranulation and cytokine production, whereas cytokine-induced or antigen-experienced memory-like NK cells depend more on oxidative phosphorylation (OXPHOS), fatty acid oxidation, and mitochondrial biogenesis to sustain long-term activity (3, 4). Mitochondria are dynamic organelles that coordinate metabolism, differentiation, cell death, and autophagy, and their function is maintained by a balance between fusion and fission in response to environmental cues (5). Fusion, mediated by mitofusins (MFN1 and MFN2) and optic atrophy 1 (OPA1), promotes interconnected mitochondrial networks that support mitochondrial oxidative capacity and cell survival, whereas fission, regulated by dynamin-related protein 1 (Drp1), is linked to mitophagy and mitochondrial quality control (6, 7, 8). Disruption of these metabolic and mitochondrial programs impairs lymphocyte survival, proliferation, and effector activity, particularly under pathological conditions such as tumors and chronic viral infections, where nutrient restriction, hypoxia, and mitochondrial dysfunction further compromise the generation and maintenance of memory-like NK cells (4, 9, 10). Consequently, inappropriate metabolic reprogramming is now recognized as a key driver of immune dysfunction in the tumor and inflammatory microenvironment. Accordingly, clinical NK cell products such as cytokine-induced memory-like NK cells and CAR-NK cells likewise require preserved mitochondrial fitness and metabolic flexibility to achieve durable anti-tumor responses (11, 12, 13).

Inhibitory receptors are increasingly recognized as key upstream regulators of lymphocyte metabolism. For example, engagement of the checkpoint receptor PD-1 on T cell constrains glycolytic activity and mitochondrial function, promoting a metabolically exhausted state that limits effector differentiation while favoring long-lived but functionally restrained cells (14, 15). Similar principles likely apply to NK cells, in which ligand-induced signaling through inhibitory receptors can likewise reshape energetic pathways and influence their proliferation, survival, and cytotoxic capacity.

Among the numerous surface receptors abundantly expressed on NK cells, sialic acid-binding immunoglobulin-like lectin (Siglec-7) has been validated as a glyco-immune checkpoint and represents a promising target for cell-based immunotherapy (16, 17). Structurally, Siglec-7 comprises an extracellular N-terminal V-set immunoglobulin-like domain responsible for sialic acid recognition, two C2-set immunoglobulin-like domains, a single transmembrane region, and a cytoplasmic tail containing two immunoreceptor tyrosine-based inhibitory motifs (ITIMs) (18). Through these ITIMs, Siglec-7 recruits SHP-1 and SHP-2 phosphatases to dampen activating signals in response to sialylated glycans overexpressed on tumor or infected cells (19). Its expression influences key physiological functions; notably, altered Siglec-7 on NK cells in the context of viral infection or chronic inflammatory states such as obesity is closely associated with NK cell dysfunction (20, 21, 22). Beyond NK cells, Siglec-7 has been identified on other immune and hematopoietic cell types, including macrophages, CD8^+^ T cells, and platelets (23, 24, 25). Of note, Siglec-7 has recently been recognized as an important marker of memory CD8^+^ T cells, and its expression level is closely associated with both metabolic capacity and cellular functional fitness. Moreover, recent work from megakaryocytes (MKs) implicates Siglec-7 in regulating mitochondrial organization and function during maturation and platelet production, suggesting that this receptor may couple glycan sensing to mitochondrial dynamics across hematopoietic lineages (26).

Despite these observations, it remains unclear whether Siglec-7 expression and engagement are associated with mitochondrial dynamics and metabolism in NK cells, including whether Siglec-7^NEG^ and Siglec-7^POS^ subsets differ in their reliance on glycolysis *versus* OXPHOS and in related mitochondrial properties. In this study, we first compared the effects of metabolic inhibition on NK degranulation between Siglec-7^NEG^ and Siglec-7^POS^ subsets, and then examined mitochondrial organization, membrane potential, and bioenergetic parameters in relation to Siglec-7 expression and following ligation with an agonistic anti-Siglec-7 antibody. Our findings identify distinct mitochondrial and metabolic features associated with Siglec-7 expression and antibody-mediated Siglec-7 ligation, including altered mitochondrial dynamics, reduced proliferative capacity, and increased apoptotic susceptibility. These observations provide a basis for further investigation into Siglec-7-associated mitochondrial pathways and their potential relevance to NK cell-based immunotherapy.

## Results

### Metabolic inhibition more strongly reduced degranulation in Siglec-7^POS^ NK cells

Previous studies have demonstrated that the cytotoxic function of NK cells relies on glycolysis (27). We first examined how metabolic inhibition affects degranulation in Siglec-7-defined NK cell subsets. Degranulation (CD107a) was measured in peripheral blood NK cells from healthy donors co-cultured with K-562 target cells in the presence of the glycolysis inhibitor 2-DG and OXPHOS inhibitor oligomycin. Both inhibitors reduced degranulation in bulk NK cells compared with the control condition (Fig. 1*A*). When degranulation was stratified by Siglec-7 expression, 2-DG and oligomycin impaired CD107a response in both Siglec-7^NEG^ and Siglec-7^POS^ PBNK subsets (Fig. 1, B and C). To compare the effects of metabolic inhibition between these subsets, we examined their degranulation responses under control, 2-DG, and oligomycin conditions. In the donor set summarized in Figure S1*A*, both 2-DG and oligomycin reduced degranulation in Siglec-7^NEG^ and Siglec-7^POS^ NK cells, with a greater reduction observed in the Siglec-7^POS^ subset. To corroborate these findings in a cell-line model, we analyzed the NK-92MI cell, in which a fraction of the population expressed surface Siglec-7 (Fig. S2). Metabolic inhibition decreased CD107a degranulation in bulk NK-92MI cells and in both Siglec-7^NEG^ and Siglec-7^POS^ NK-92MI subsets (Fig. 1, D–F). Oligomycin-associated inhibition of degranulation was greater in Siglec-7-expressing NK-92MI cells than in Siglec-7^NEG^ cells (Fig. S1*B*).

**Figure 1:**
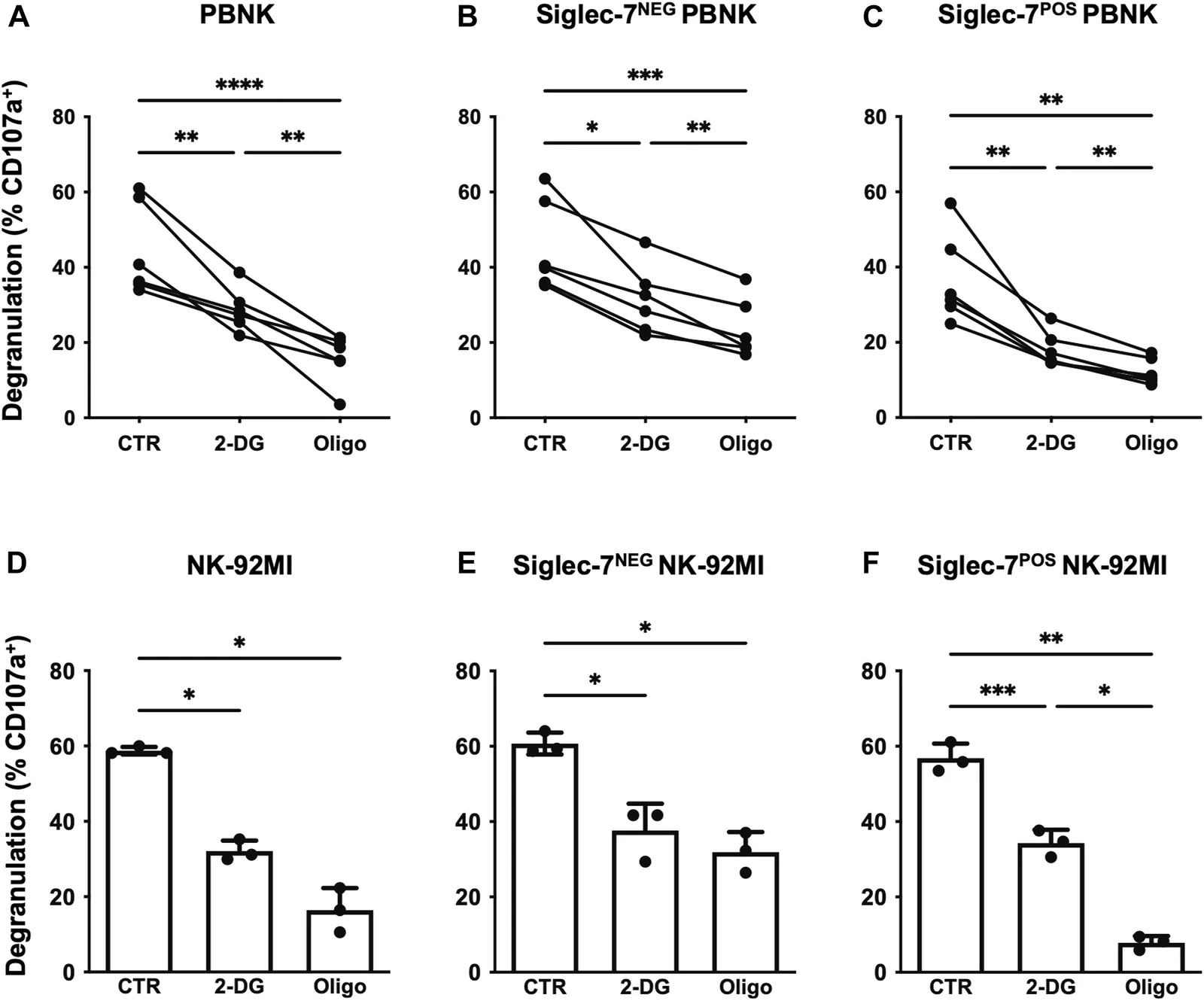
**Degranulation response of Siglec-7^NEG^ and Siglec-7^POS^ NK cells to metabolic inhibition**. CD107a degranulation of bulk (*A*, *D*), Siglec-7^NEG^ (*B*, *E*), and Siglec-7^POS^ (*C*, *F*) peripheral blood NK cells (PBNK; *A*–*C*) and NK-92MI (*D*–*F*) co-cultured with K-562 target cells under control (CTR), 2-DG-treated, or oligomycin (Oligo) treatment at a 1:1 effector-to-target (E:T) ratio for 1 h. Data are presented as mean ± SD of biological replicates (n = 6 independent donors for PBNK in *A*–*C*, n = 3 independent NK-92MI experiments in *D*–*F*). Statistics significance was determined by one-way ANOVA followed by Tukey’s multiple-comparison test; ∗*p* < 0.05, ∗∗*p* < 0.01, ∗∗∗*p* < 0.001, ∗∗∗∗*p* < 0.0001.

### Siglec-7^NEG^ NK cells exhibit greater mitochondrial mass and a more fused morphology

Given that OXPHOS inhibition exerted a stronger suppressive effect on cytotoxicity in Siglec-7-defined NK cell subsets than glycolytic blockade, we next compared mitochondrial mass between Siglec-7^NEG^ and Siglec-7^POS^ NK cells. MitoTracker Green FM staining revealed greater mitochondrial mass in Siglec-7^NEG^ NK cells than in Siglec-7^POS^ counterparts in both peripheral blood NK cells and NK-92MI cells (Fig. 2, *A* and *B*).

**Figure 2:**
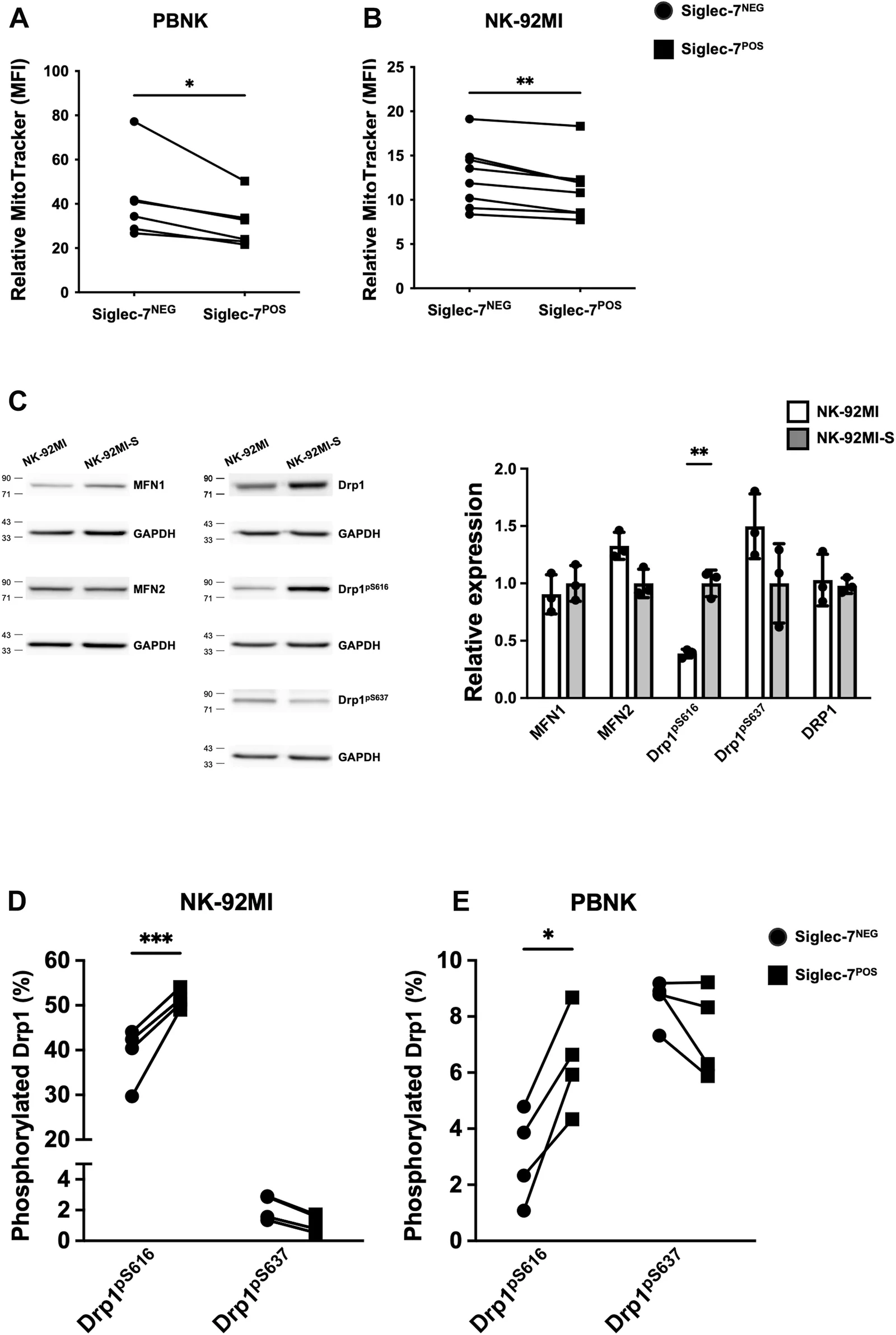
**Reduced mitochondrial mass and increased Drp1 Ser^616^ phosphorylation in Siglec-7-expressing NK cells**. *A* and *B*, relative MitoTracker mean fluorescence intensity (MFI) of Siglec-7NEG and Siglec-7POS subsets in PBNK (*A*) and NK-92MI cells (*B*). *C*, representative immunoblots of MFN1, MFN2, Drp1, phospho-Drp1^Ser616^ (Drp1^pSer616^), and phospho-Drp1^Ser637^ (Drp1^pSer637^) in NK-92MI and NK-92MI-S cells, with GAPDH as loading control (*left*). The right panel shows quantification of band intensities normalized to GAPDH and expressed relative to NK-92MI. *D* and *E*, percentage of phosphorylated Drp1 at Ser616 and Ser637 in Siglec-7NEG and Siglec-7POS subsets of NK-92MI cells (*D*) and in PBNK (*E*). Each dot represents one donor (*A*, *E*) or one independent experiment (*B, D*). Data are presented as mean ± SD of 3 to 8 independent experiments [n = 6 for (*A*), n = 8 for (*B*), n = 3 for (*C*–*E*)]. Statistical significance was calculated using paired t-tests; ∗*p* < 0.05, ∗∗*p* < 0.01, ∗∗∗*p* < 0.001.

Mitochondrial dynamics regulate cellular function through opposing fusion and fission processes (7). To examine mitochondrial dynamics in relation to Siglec-7 expression, we analyzed NK cells with differential Siglec-7 expression. The NK-92MI-S subline, derived from NK-92MI cells in our laboratory, exhibited approximately 90% of cells were surface-positive for Siglec-7 (Fig. S2) (28). Immunoblotting revealed comparable total levels of the fusion regulators MFN1, MFN2, and the fission regulator Drp1 between the two lines (Fig. 2*C*). However, NK-92MI-S displayed increased pro-fission Drp1 phosphorylation of Ser^616^, an activating site, but not at Ser^637^, an inhibitory site (Fig. 2*C*). To directly compare the phosphorylation at these two sites between Siglec-7^NEG^ and Siglec-7^POS^ NK cells, we next performed phospho-specific flow cytometry. In both models, Siglec-7^POS^ cells consistently exhibited higher levels of Drp1 Ser^616^ phosphorylation, whereas no corresponding difference was observed at Ser^637^ (Fig. 2, *D* and *E*). Taken together, these data indicate that Siglec-7-expressing NK cells display a pro-fission profile characterized by increased Drp1 Ser^616^ phosphorylation in both cell-line and primary-NK-cell models.

To directly assess mitochondrial morphology, we performed confocal microscopy of NK-92MI cells stained with Hoechst 33,342, MitoTracker Green FM, and Siglec-7 antibody. Siglec-7^NEG^ NK (yellow arrow) exhibited a greater mitochondrial signal than Siglec-7^POS^ cells (white arrow) (Fig. 3*A*). ImageJ quantification of mitochondrial morphometric parameters, including area, perimeter, form factor, aspect ratio, branch length, branch junction number, branch end point number, and mitochondrial count, indicated that Siglec-7^NEG^ NK cells had larger and more interconnected mitochondrial networks than Siglec-7^POS^ cells (Fig. 3, *B* and *C*). Together with the FACS data, these findings indicate that Siglec-7^NEG^ NK cells exhibit fewer pro-fission features and a more fused mitochondrial morphology than Siglec-7^POS^ cells (Figs. 2 and 3, *A*–*C*).

**Figure 3:**
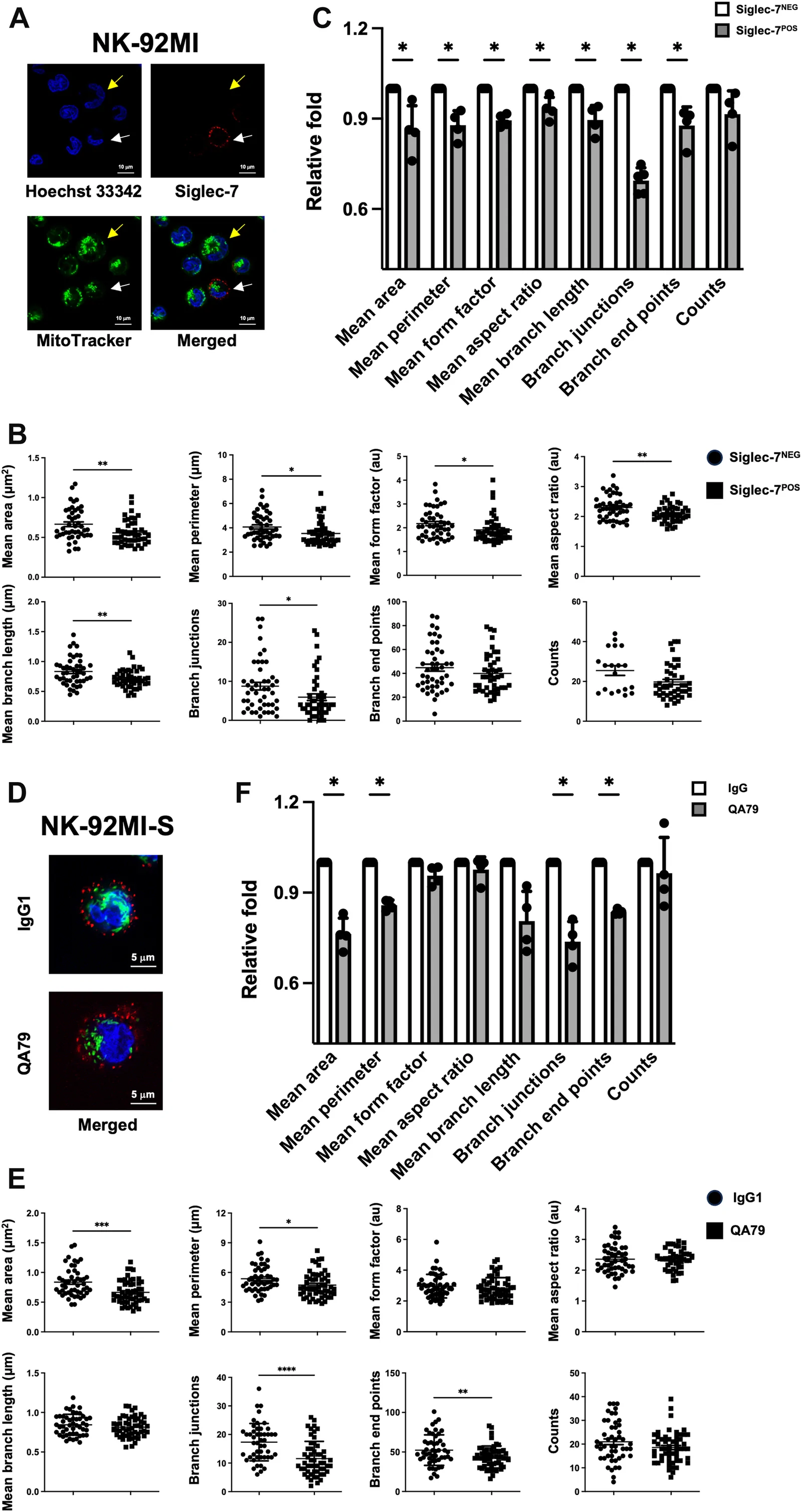
**Siglec-7 expression and engagement alter mitochondrial morphology in NK cell lines**. *A*, representative confocal images of NK-92MI cells stained with Hoechst 33,342 (*blue*), anti-Siglec-7 (*red*), and MitoTracker Green FM (*green*), shown as individual channels and merged. Yellow and white arrows indicate Siglec-7^NEG^ and Siglec-7^POS^ cells, respectively. *B*, quantification of mitochondrial morphology parameters, including mean area, mean perimeter, mean form factor, mean aspect ratio, mean branch length, branch junction, branch end point, and mitochondrial count in Siglec-7^NEG^ and Siglec-7^POS^ NK-92MI cells. *C* and *F*, summary bar graphs showing the relative fold changes in mitochondrial morphology parameters between Siglec-7^NEG^ and Siglec-7^POS^ NK-92MI (*C*) and NK-92MI-S (*F*) cells from three independent experiments. (*D*) Representative confocal images of NK-92MI-S cells treated with isotype control IgG1 or anti-Siglec-7 antibody (QA79), stained as shown in (*A*). *E*, quantification of mitochondrial morphology parameters in NK-92MI-S cells treated with IgG1 or QA79, as described in (*B*). Data are presented as mean ± SD and statistical significance was calculated using unpaired t-tests; ∗*p* < 0.05, ∗∗*p* < 0.01, ∗∗∗*p* < 0.001.

### QA79-mediated Siglec-7 ligation promotes mitochondrial fission

To examine the effects of Siglec-7 ligation on mitochondrial morphology, NK-92MI-S cells were treated for 1 h with agonistic anti-Siglec-7 antibody QA79 or the corresponding isotype control, followed by immunofluorescence staining and confocal image analysis (Fig. 3*D*). Siglec-7 engagement reduced mitochondrial size and altered morphology in NK-92MI-S cells, as evidenced by significant decreases in mean area, perimeter, branched junctions, and branched end points (Fig. 3, *E* and *F*). In parallel control experiments, sodium azide was titrated at concentrations matching or exceeding those present in the QA79 preparations and did not measurably affect MitoTracker or TMRE fluorescence in NK-92MI cells, indicating that preservative concentrations used did not perturb mitochondrial mass or membrane potential (Fig. S3). Consistent with findings in NK-92MI-S, QA79 stimulation of primary NK cells similarly decreased mitochondrial area, perimeter, form factor, and branch junctions (Fig. 4), collectively indicating that Siglec-7 engagement promotes a mitochondrial morphology consistent with increased fission across both models. Supporting this interpretation, quantitative morphometric analysis of PBNK showed that QA79-induced changes were most evident in Siglec-7-expressing cells, whereas minimal effects were observed in Siglec-7^NEG^ cells (Fig. S4).

**Figure 4:**
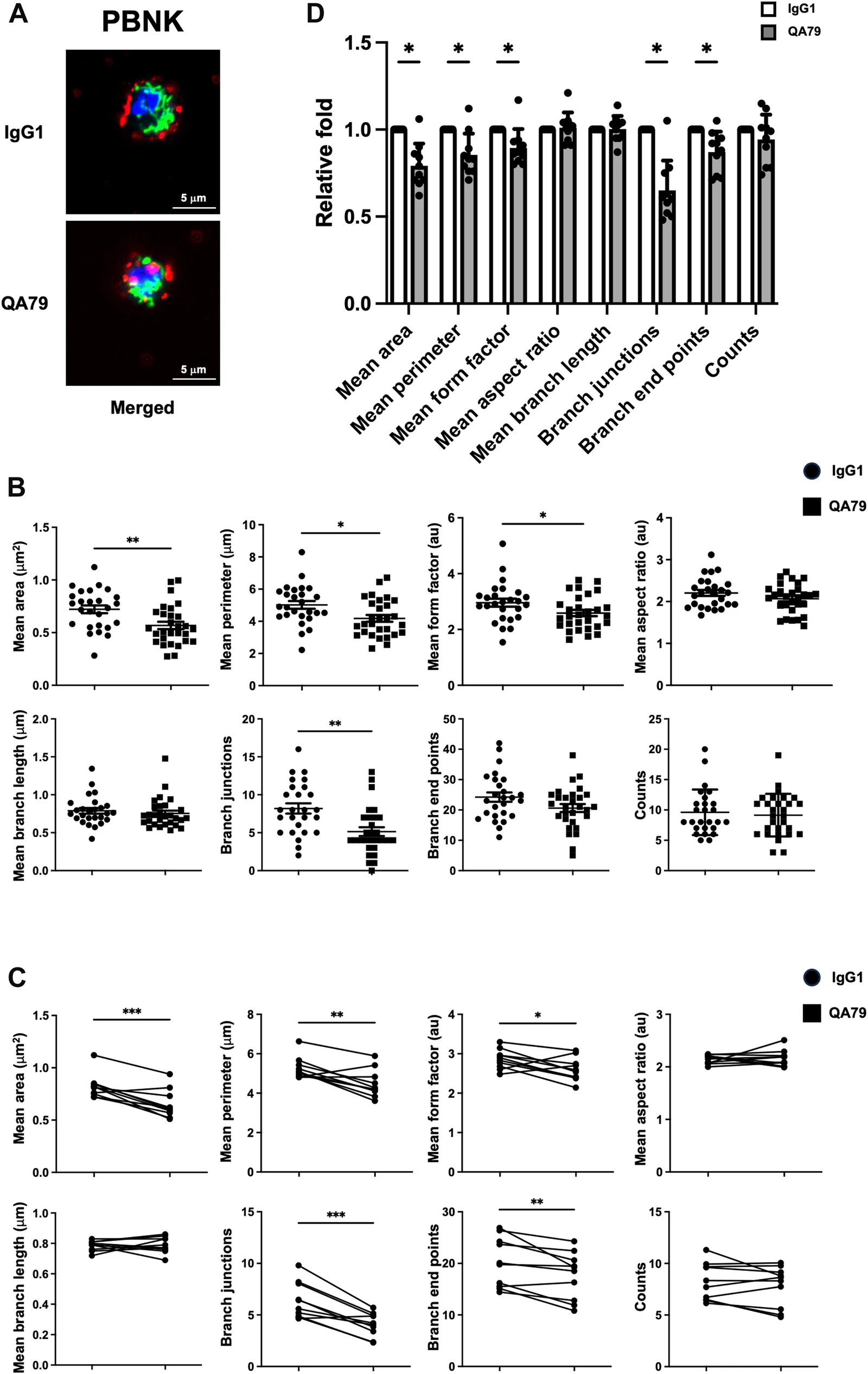
**Siglec-7 ligation alters mitochondrial morphology in peripheral NK cells**. *A*, representative confocal images of PBNK treated with isotype control IgG1 or anti-Siglec-7, stained as Figure 3*A*. *B*–*D*, quantification of mitochondrial morphology parameters in PBNK stimulated with IgG1 or QA79. Each dot and line represent one cell (*B*) or one donor (*C*, *D*), respectively. Data are presented as mean ± SD [n = 10 for (*C*, *D*)]. Statistical significance was calculated using unpaired t-tests (*B*) and paired t-tests (*C*, *D*); ∗*p* < 0.05; ∗∗*p* < 0.01, ∗∗∗*p* < 0.001.

### Distinct metabolic profiles associated with Siglec-7 expression and QA79 ligation in NK cells

To assess the metabolic differences associated with Siglec-7 expression, we measured ECAR and OCR in NK-92MI (∼85% Siglec-7^NEG^) and NK-92MI-S cells (∼90% Siglec-7^POS^) (Fig. S2), using these measures to assess glycolytic activity and mitochondrial respiration, respectively. No significant differences in glycolytic parameters were observed between the two lines (Fig. 5*A*). In contrast, NK-92MI-S cells exhibited significantly higher basal respiration, maximal respiration, ATP production, spare respiration capacity, and proton leak than NK-92MI cells (Fig. 5*B*), indicating greater mitochondrial respiratory capacity in NK-92MI-S cells under the conditions tested.

**Figure 5:**
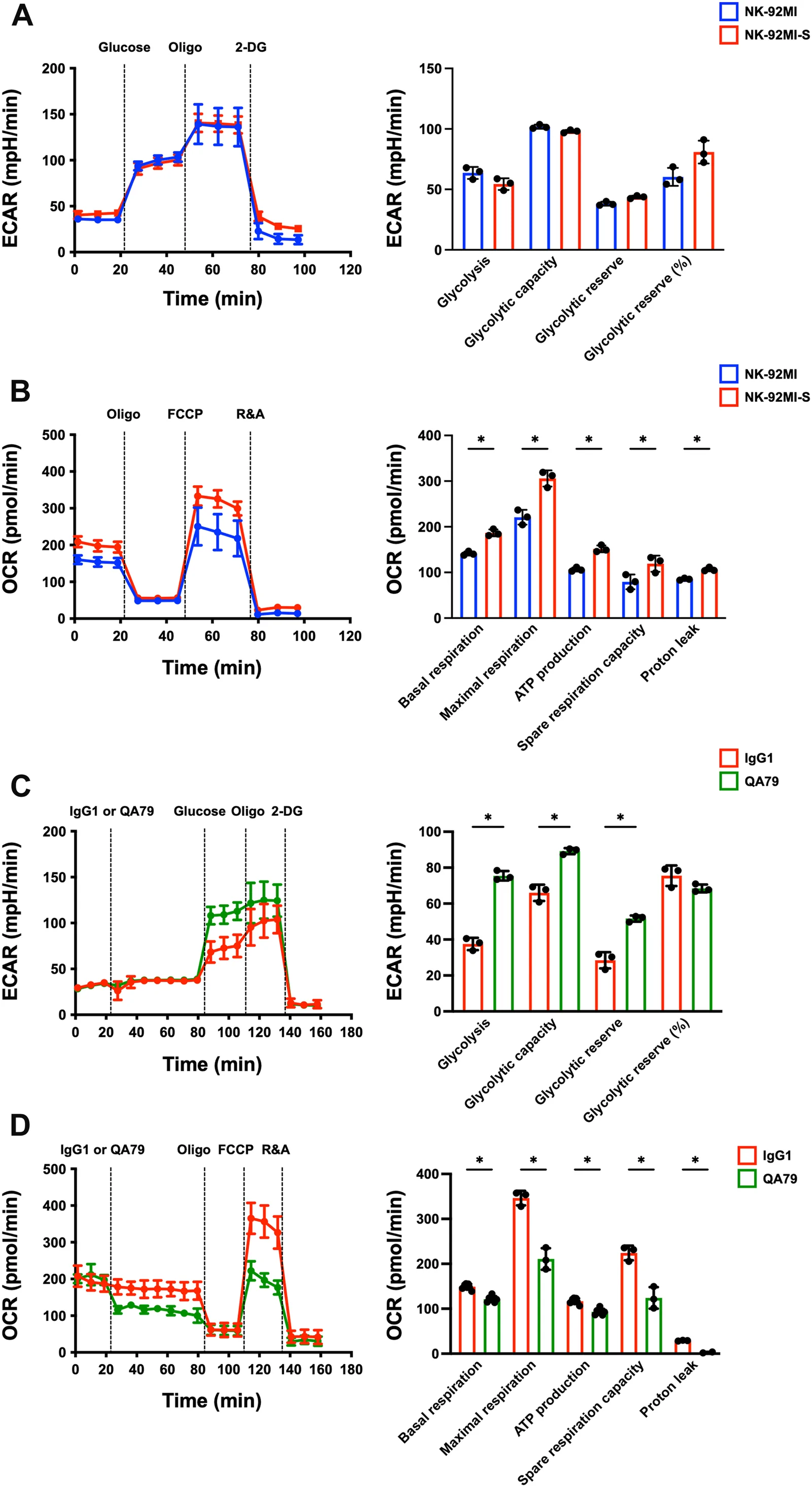
**Siglec-7 expression and engagement change glycolytic and mitochondrial respiratory capacity in NK cells**. *A*, analyses of ECAR in NK-92MI and NK-92MI-S cells. ECAR of NK cells with sequential addition of glucose (10 mM), the ATP synthase inhibitor oligomycin (Oligo) (10 mM) and glucose analog 2-DG (50 mM) were assessed in real time. *B*, real-time changes of OCR in NK-92MI and NK-92MI-S cells with addition of Oligo (1 μM), the mitochondrial uncoupler FCCP (1 μM), and inhibitors of mitochondrial electron transport chain complex I and III, rotenone (0.5 μM) and antimycin A (0.5 μM) (R&A), were measured. *C*, ECAR traces (*left*) and quantification of glycolytic parameters (*right*) in NK-92MI-S cells pre-treated with isotype control IgG1 or QA79, followed by sequential injections of glucose, oligomycin, and 2-DG. *D*, OCR traces (*left*) and quantification of mitochondrial respiration parameters (*right*) in NK-92MI-S cells pre-treated with IgG1 or QA79, followed by sequential injections of oligomycin, FCCP, and R&A. For all panels, traces show a representative independent experiment and bar graphs show mean ± SD of biological replicates (n = 3 independent Seahorse experiments), each experiments included four technical wells per condition. Statistical significance was determined using paired t-tests; ∗*p* < 0.05.

We next examined metabolic responses to Siglec-7 ligation by stimulating NK-92MI-S cells with anti-Siglec-7 antibody QA79 for 1 h. Compared with isotype control, QA79 significantly increased ECAR-derived parameters, including glycolysis, glycolytic capacity, and glycolytic reserve (Fig. 5*C*), while the percentage of glycolytic reserve remained unchanged. Conversely, Siglec-7 treatment significantly reduced OCR-derived parameters, including basal respiration, maximal respiration, ATP production, spare respiration capacity, and proton leak (Fig. 5*D*). Together, these data indicate that QA79-mediated Siglec-7 ligation is associated with enhanced glycolytic parameters and reduced mitochondrial parameters in NK-92MI-S cells, consistent with a dual metabolic shift following Siglec-7 ligation. In support of these findings, analysis of NK-92MI and NK-92MI-S cells showed that QA79 markedly reduced OCR-derived respiratory parameters in Siglec-7^high^ NK-92MI-S cells, whereas OCR-derived parameters in Siglec-7^low^ NK-92MI cells were similar following QA79 and IgG1 treatment (Fig. S5).

### Siglec-7 expression and QA79 ligation are associated with differences in mitochondrial membrane potential in NK cells

During OXPHOS, proton pumps in the inner mitochondrial membrane generate mitochondrial membrane potential (ΔΨm), which is tightly linked to and essential for mitochondrial respiration. To measure ΔΨm, we used the fluorescent dye TMRE, which is sensitive to changes in membrane potential. In NK-92MI cells, basal TMRE fluorescence was significantly higher in Siglec-7^NEG^ cells than in Siglec-7^POS^ cells, indicating a more hyperpolarized ΔΨm in this subset (Fig. 6, *A* and *B*). Upon FCCP addition, the TMRE signal was markedly reduced in both subsets, and TMRE fluorescence converged to similar levels, consistent with FCCP acting as a negative control that collapses ΔΨm (Fig. 6, *B* and *C*). A similar pattern of higher basal TMRE followed by a FCCP-induced loss of signal was observed in peripheral NK cells (Fig. 6, *D*–*F*). Collectively, these data indicate that Siglec-7^NEG^ cells maintain a more hyperpolarized ΔΨm at baseline than Siglec-7^POS^ cells, consistent with their higher mitochondrial mass and fused mitochondrial network rather than necessarily reflecting higher steady-state OXPHOS flux *per se* (Figs. 2, *A* and *B* and 3, *A* and *B*).

**Figure 6:**
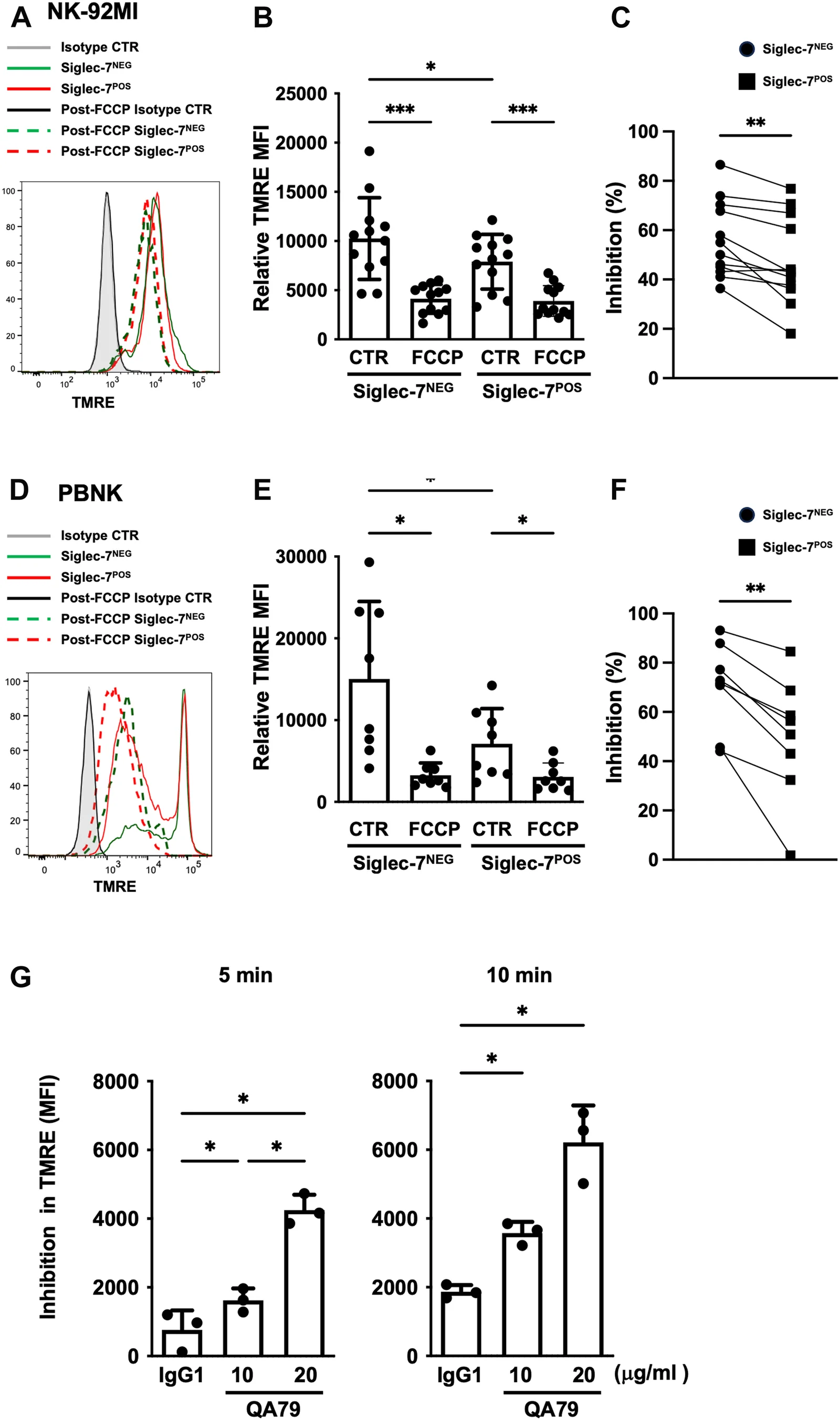
**QA79-mediated Siglec-7 ligation is associated with decreases in mitochondrial membrane potential in NK cells**. *A* and *D*, representative TMRE fluorescence histograms of Siglec-7^NEG^ (*green lines*) and Siglec-7^POS^ (*red lines*) NK-92MI cells (*A*) and PBNK (*D*) before (*solid lines*) and after FCCP treatment (*dashed lines*). *B* and *E*, relative TMRE MFI values normalized to unstained controls and are shown as mean ± SD [n = 12 for (*B*), n = 8 for (*E*)]. *C* and *F*, TMRE inhibition percentage by FCCP in Siglec-7^NEG^ and Siglec-7^POS^ NK-92MI (*C*) and PBNK (*F*) is shown, calculated as the percentage decrease in TMRE MFI upon FCCP treatment. *G*, inhibition of TMRE fluorescence intensity in NK-92MI-S cells stimulated with IgG1 or QA79 (10 or 20 μg/ml) for 5 min (*left*) or 10 min (*right*), measured by plate reader assay. Fluorescence values were corrected for background signal from unstained controls and expressed relative to T = 0 (n = 3 independent experiments with six technical replicates per group). Data are represented as mean ± SD. Statistical significance was determined using one-way ANOVA followed by Tukey’s multiple-comparison test (*B*, *E*, *G*) and paired t-tests (*C*, *F*); ∗*p* < 0.05, ∗∗*p* < 0.01, ∗∗∗*p* < 0.001.

To examine the effect of QA79-mediated Siglec-7 ligation on ΔΨm, changes in TMRE fluorescence were quantified using a plate reader following QA79 treatment at different doses and time points. QA79 engagement in NK-92MI-S cells significantly decreased ΔΨm at both 5 and 10 min in a dose-dependent manner (Fig. 6*G*). In agreement with this, TMRE histograms of NK-92MI cells gated into Siglec-7^NEG^ and Siglec-7^POS^ populations showed that QA79 reduced TMRE fluorescence mainly in Siglec-7^POS^ cells, whereas minimal changes were observed in the Siglec-7^NEG^ subset (Fig. S6). Taken together, these results show that QA79-mediated Siglec-7 ligation was associated with a transient reduction in ΔΨm in NK cells, with the effect observed predominantly in Siglec-7^POS^ cells.

### Autophagy, proliferation, and survival differ between Siglec-7^NEG^ and Siglec-7^POS^ subset in NK cells

Subsequently, we compared autophagy- and mitophagy-related proteins in NK-92MI and NK-92MI-S cells. Immunoblotting revealed similar levels of autophagy-related protein 5 (ATG5), microtubule-associated protein 1A/1B-light chain 3B-I (LC3B-I), and PARKIN between the two lines, whereas LC3B-II and sequestosome one (p62) tended to be increased in NK-92MI-S cells (Fig. 7*A*). These data suggest that overall autophagy/mitophagy machinery is largely preserved, with selective LC3B-II accumulation suggesting increased autophagosome formation in Siglec-7^POS^ NK cells. We next assessed autophagic vacuoles in NK cells stratified by Siglec-7 expression. In both NK-92MI and peripheral blood NK cells, Siglec-7^POS^ cells showed higher frequencies of CYTO-ID^POS^ cells than their Siglec-7^NEG^ counterparts, suggesting increased accumulation of autophagic vacuole-containing compartments (Figs. 7, *B* and *C* and Fig. S7, *B*–*C*). These findings indicate that Siglec-7 expression is associated with an increased proportion of autophagic vacuole-positive cells.

**Figure 7:**
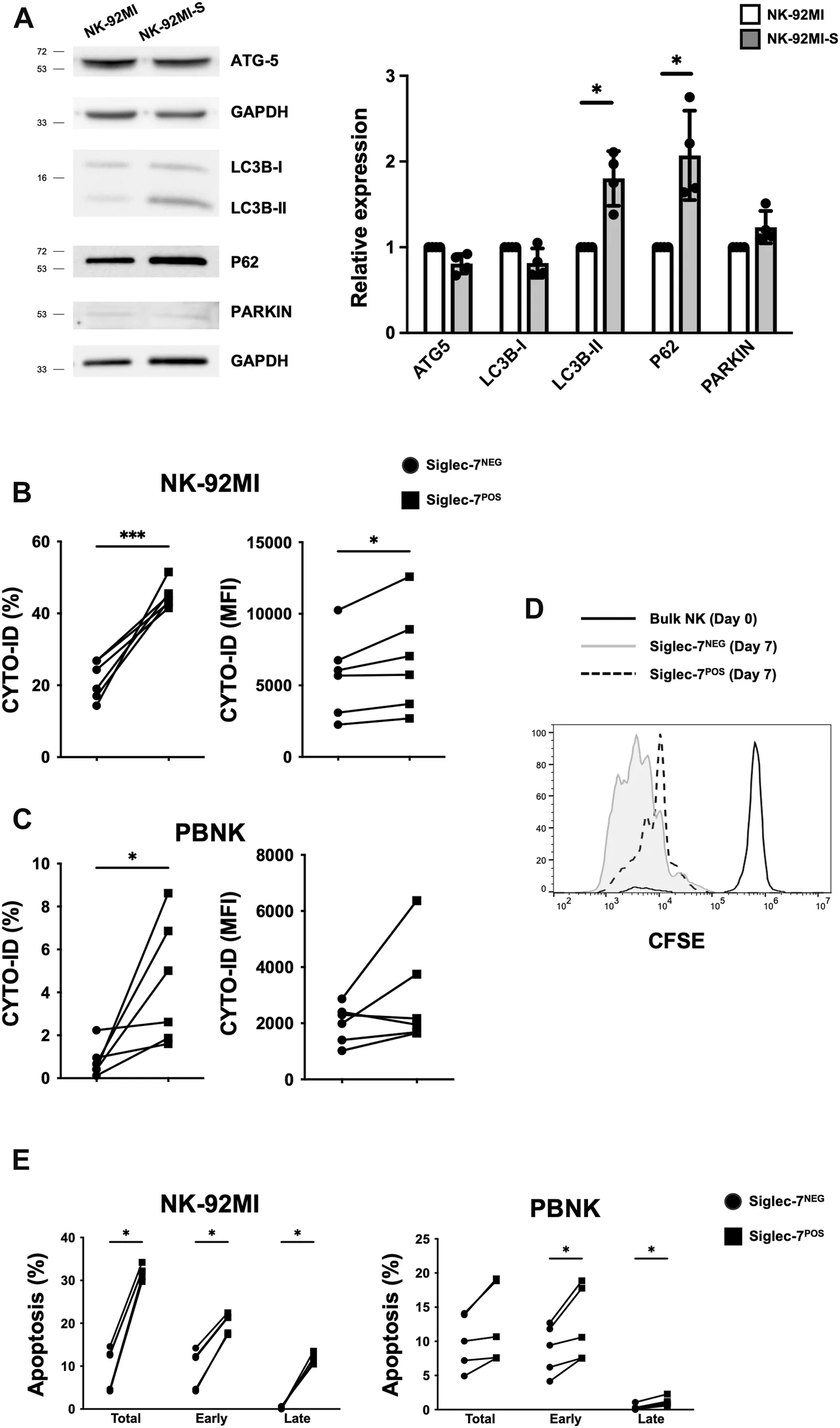
**Comparison of autophagic vacuole accumulation, proliferation, and apoptosis between Siglec-7^NEG^ and Siglec-7^POS^ NK cells**. *A*, immunoblot analysis of ATG5, LC3B-I, LC3B-II, p62, and PARKIN in NK-92MI and NK-92MI-S cells, with GAPDH as loading control (*left*). Bar graphs show quantification of band intensities normalized to GAPDH and expressed relative to NK-92MI (right). *B, C*, CYTO-ID staining of autophagic vacuoles in NK-92MI (*B*) and PBNK (*C*) stratified by Siglec-7 expression. Frequencies of CYTO-ID-positive cells (*left*) and relative CYTO-ID MFI (*right*) within the Siglec-7^NEG^ and Siglec-7^POS^ subsets are shown. *D*, CFSE dilution profiles of bulk cells at Day 0 (*solid black line*), and Siglec-7^NEG^ (*solid grey line*) and Siglec-7^POS^ (*dashed black line*) PBNK at Day 7, illustrating the proliferation of each subset over 7 days of IL-2 plus IL-15 culture. Data are represented one of four independent experiments. *E*, apoptosis of NK-92MI (*left*) and PBNK (*right*) stratified by Siglec-7 expression, assessed by Annexin and 7-AAD staining. Frequencies of total, early, and late apoptotic cells are shown for Siglec-7^NEG^ and Siglec-7^POS^ cells. Data are represented as mean ± SD [n = 4 for (*A*), n = 6 for (*B*, *C*), n = 5 for (*E*)]. Statistical significance was determined using paired t-tests; ∗*p* < 0.05, ∗∗∗*p* < 0.001.

We next examined the proliferative capacity of Siglec-7-defined subsets. Enriched PBNK cells were labeled with CFSE at day 0 and cultured for 7 days with IL-2 plus IL-15, and CFSE dilution was analyzed using the Proliferation platform in FlowJo. Under these conditions, Siglec-7^NEG^ NK cells consistently displayed higher division and expansion indices than Siglec-7^POS^ cells; for example, the division index and expansion indices of Siglec-7^NEG^ cells averaged approximately 3.7 and 21.8, respectively, compared with 3.4 and 15.1 for Siglec-7^POS^ cells (Figs. 7*D* and Fig. S8). To assess cytokine dependence, we also analyzed CFSE dilution under IL-15 alone, which similarly supported robust proliferation in both subsets while Siglec-7^NEG^ NK cells retained higher proliferation- and expansion-related indices than Siglec-7^POS^ NK cells (Fig. S8). These data indicate that Siglec-7^NEG^ NK cells undergo more rounds of cell division and achieve greater proliferative expansion than the Siglec-7^POS^ subset. Finally, we evaluated apoptosis in NK-92MI and primary peripheral NK cells using Annexin V/7-AAD staining. In NK-92MI cells, Siglec-7^POS^ cells exhibited significantly higher frequencies of total, early, and late apoptotic cells than Siglec-7^NEG^ cells (Fig. 7*E*). A similar pattern, with significantly elevated early and late apoptosis in Siglec-7^POS^ subset, was observed in primary NK cells (Fig. 7*E*). Collectively, these data indicate that Siglec-7 expression in NK cells is associated with enhanced autophagic vacuole accumulation, reduced proliferative capacity, and heightened apoptotic susceptibility.

## Discussion

In this study, we delineate a role for the glyco-immune checkpoint receptor Siglec-7 in coupling mitochondrial dynamics and metabolic state to human NK cell effector function in both cell lines and primary cells. Analysis of Siglec-7^NEG^ and Siglec-7^POS^ subsets showed that inhibition of glycolysis and oxidative phosphorylation compromised degranulation in both subsets, with 2-DG and oligomycin generally exerting stronger inhibitory effects on Siglec-7^POS^ cells (Figs. 1 and Fig. S1), consistent with divergent metabolic wiring between these populations. Siglec-7^NEG^ NK cells exhibit greater mitochondrial mass and a more fused morphology (Figs. 2, *A* and *B* and Fig. 3, *A*–*C*), whereas QA79-mediated Siglec-7 ligation was associated with mitochondrial fission (Figs. 3, *D*–*F* and Fig. 4), reduced ΔΨm (Fig. 6), and altered mitochondrial dynamics. Furthermore, Siglec-7 expression was associated with differences in autophagy, proliferation, and survival (Fig. 7), consistent with a relationship between Siglec-7 expression, mitochondrial stress, and NK metabolic and survival states. Following QA79-mediated Siglec-7 ligation, ΔΨm decreased rapidly, as evidenced by TMRE staining (Fig. 6*G*). Whether this transient decrease contributes to subsequent mitophagic or autophagic responses and increased apoptotic susceptibility in Siglec-7^POS^ NK cells remains to be determined (Fig. 7). By contrast, Siglec-7^NEG^ peripheral NK cells were more adaptable to inhibition of both glycolysis and OXPHOS, enabling them to sustain stronger cytotoxic responses under metabolically stressful conditions (Fig. 1*A*).

Although glycolysis has been widely implicated as the predominant metabolic pathway supporting NK degranulation and cytotoxicity (27, 29), our data showed that oligomycin-mediated inhibition of OXPHOS suppressed CD107a degranulation to a greater extent than 2-DG treatment in peripheral NK cells (Fig. 1*A*). This finding is consistent with previous reports demonstrating that primary NK cell cytotoxicity is dependent on OXPHOS (3, 30), and suggests that glycolysis and OXPHOS are functionally coupled in supporting the rapid energy demands of granule exocytosis. Together, these findings indicate that both glycolysis and OXPHOS contribute to NK cell degranulation and that inhibition of either pathway is sufficient to impair cytotoxic responses. The relative contribution of each pathway may vary depending on the cellular context, physiological state, and nature of the activating stimulus, yet these two pathways act in a coordinated rather than mutually exclusive manner to sustain NK cell cytotoxicity.

Notably, QA79-induced depolarization of ΔΨm was evident at 5 to 10 min (Fig. 6*G*), indicating a rapid response to QA79-mediated Siglec-7 ligation. This change ΔΨm occurred alongside the dual metabolic shift observed in our experiment, namely enhanced glycolytic capacity alongside broad suppression of mitochondrial OXPHOS (Fig. 5, C and D). Together, these findings suggest that QA79 treatment was associated with increased glycolytic parameters and reduced mitochondrial respiratory activity in NK cells under the conditions tested, which may contribute to the attenuation of NK cell cytotoxic responses (Fig. 1). Consistent with prior studies identifying Siglec-7 as a ligand-dependent inhibitory checkpoint on NK cells, our data show that Siglec-7^POS^ NK cells display a mitochondrial stress-associated phenotype under isotype control conditions, whereas QA79 treatment is associated with additional acute changes in mitochondrial membrane potential and metabolic parameters (31, 32, 33). The present study does not define the downstream pathway responsible for the metabolic responses observed following QA79 treatment. Further genetic, ligand-blocking, and pathway-specific studies will be required to establish Siglec-7 dependence and define the responsible mechanism.

In this study, we found that Siglec-7^NEG^ peripheral NK cells expanded preferentially under both IL-15 alone and IL-2 plus IL-15 stimulation, consistent with subset-specific differences in cytokine responsiveness or activation thresholds (Figs. 7*D* and Fig. S8). IL-15-mediated signaling sustains JAK1/JAK3-STAT5 and PI3K-AKT-mTOR pathways, which support NK cell survival and proliferation (34, 35, 36). The preferential expansion of Siglec-7^NEG^ NK cells under these cytokine conditions is consistent with the concept that cytokine responsiveness is tightly coupled to metabolic fitness of NK subsets (34) and suggest that Siglec-7^NEG^ NK cells may be better positioned to translate cytokine signals into robust proliferative and survival responses. Although the precise mechanisms underlying this subset-specific advantage remain to be elucidated, these findings highlight the interplay between NK cell heterogeneity and metabolic fitness, and suggest that further studies of metabolic pathways may help optimize cytokine-driven expansion of selected NK subsets for therapeutic applications.

Our findings extend current evidence linking immune function to metabolism and mitochondrial quality control. In T cells, engagement of the inhibitory receptor PD-1 exemplifies how surface receptors can reprogram cellular metabolism and thereby influence functional fate, as PD-1 signaling constrains glycolytic activity and effector differentiation (15). Similar principles govern the transition from short-lived to long-lived NK cell responses, where coordinated changes in glycolysis, OXPHOS, and fatty acid oxidation support distinct effector and memory-like states (3, 15, 37). Mitochondrial dynamics and mitophagy together constitute a quality-control system that maintains polarized, high-capacity mitochondria required for sustained cytotoxic function (3, 38). Our data extend these concepts by showing that differences in Siglec-7 expression are associated with distinct metabolic programs and mitochondrial architectures in NK cells (Fig. 2, *A* and *B* and Figure 3, Figure 4, Figure 5), suggesting an association between Siglec-7 expression, metabolic fitness and functional specialization in cytotoxic lymphocytes. Moreover, to our knowledge, our study provides the first evidence that QA79 treatment is associated with changes in OCR- and ECAR-derived metabolic parameters in NK cells.

This link between Siglec-7 status and mitochondrial competence is particularly relevant in the context of memory-like NK cells, which depend on abundant, well-functioning mitochondria and flexible metabolic programs to sustain long-term effector activity (13, 37, 38). In the absence of Siglec-7 engagement, NK cells maintained a greater oxidative capacity, as reflected by higher OCR and spare respiratory capacity in the control group compared with QA79-treated cells (Fig. 5*D*), mirroring previous findings that memory-like NK populations rely on OXPHOS to support long-term function (39, 40). These converging observations raise the possibility that Siglec-7^NEG^ NK cells are enriched for cells with memory-like bioenergetic features. Moreover, the frequency of Siglec-7^NEG^ NK cells in human peripheral blood is substantially lower than that of Siglec-7^POS^ cells (Table S1), consistent with the notion that Siglec-7^NEG^ cells may represent a relatively small subset with memory-like properties and that Siglec-7 expression status may serve as one of several phenotypic features associated with human memory-like NK cells.

In CD8^+^ T cells, Siglec-7 marks non-exhausted effector memory cells with enhanced cytotoxicity, cytokine production, and elevated glycolytic and oxidative metabolism, while locally dampening T cell receptor (TCR) signaling without globally enforcing exhaustion (24). By contrast, our data link high Siglec-7 expression in NK cells to mitochondrial fragmentation, and impaired long-term proliferation and survival (Figure 2, Figure 3 and Fig. 7, *D* and *E*). Although Siglec-7 functions as an immune checkpoint receptor in both CD8^+^ T cells and NK cells, its metabolic imprint differs between these lineages. Under steady state, Siglec-7^POS^ CD8^+^ T and NK cells both display higher OCR, indicating increased oxidative capacity, yet only Siglec-7^POS^ CD8^+^ T cells show concomitantly elevated ECAR (24), a feature not observed in Siglec-7^POS^ NK cells. Moreover, in both T and NK compartments, memory and memory-like subsets depend on OXPHOS capacity to sustain long-term function, even though their surface Siglec-7 expression levels differ markedly. This dichotomy thereby underscores the importance of Siglec-7 expression levels and cellular context in shaping lymphocyte memory and function.

Previous work from our laboratory in MK implicated Siglec-7 in regulating mitochondrial organization and function during MK maturation and platelet production, with dynamic Siglec-7 expression tracking changes in mitochondrial morphology and platelet output (26). Together with current findings in NK cells, these results suggest that Siglec-7 broadly influences mitochondrial architecture and bioenergetic programs across hematopoietic lineages. Cell type–specific outcomes are nonetheless evident, encompassing thrombopoiesis and platelet activation in MKs *versus* cytotoxic function and cellular homeostasis in NK cells. Interestingly, previous studies have implicated Siglec-7 engagement in promoting apoptosis in both NK cells and MKs, whereas Siglec-7 expressed on non-hematopoietic pancreatic β cell instead protects against apoptosis (41, 42). Collectively, these findings position Siglec-7 as a glyco-immune checkpoint with broad relevance to hematopoietic cell biology, and future studies targeting Siglec-7-associated mitochondrial pathways may offer opportunities to modulate platelet production or enhance NK cell-mediated anti-tumor immunity in clinical settings.

Our study identifies associations between Siglec-7 expression, mitochondrial organization, bioenergetic parameters, and effector-associated phenotypes in NK cells. Siglec-7^POS^ NK cells were particularly vulnerable to metabolic inhibition of glycolysis and oxidative phosphorylation, and displayed increased mitochondrial fission, altered membrane potential, and heightened autophagy, proliferation arrest and apoptosis, consistent with a metabolically vulnerable phenotype (Figs. 1–3, 6, 7). In contrast, Siglec-7^NEG^ NK cells maintain greater mitochondrial mass with a fused morphology and preserve degranulation under metabolic stress, indicative of more robust energetic capacity (Figure 1, Figure 2, Figure 3). Moreover, QA79-mediated Siglec-7 ligation was associated with reduced mitochondrial respiration parameters and enhanced glycolytic parameters in NK-92MI-S cells, consistent with context-dependent metabolic changes following antibody treatment (Fig. 5). Collectively, these findings support associations between Siglec-7 expression or antibody-mediated ligation, mitochondrial organization, bioenergetic parameters, and NK cytotoxic competence and survival. These observations provide a basis for further investigation into Siglec-7-associated mitochondrial pathways and their potential relevance to NK cell-based immunotherapy.

## Experimental procedures

### Cell culture

Human NK cell line NK-92MI and leukemia K-562 cells were purchased from Bioresource Collection and Research Center. NK-92MI and derived NK-92MI-S cells were cultured in α-MEM supplemented with 12.5% fetal bovine serum (FBS), 12.5% horse serum, 2 mM L-glutamine, 2 mM inositol, 0.1 mM 2-mercaptoethanol, 20 mM folic acid, 50 units/ml of penicillin, and 50 μg/ml of streptomycin in a humidified incubator with 5% CO_2_ at 37 ˚C (28). All reagents were purchased from Thermo Fisher Scientific.

Peripheral blood mononuclear cells (PBMCs) from healthy donors were isolated using Ficoll-Paque PLUS (GE Healthcare), and NK cells were further enriched using the EasySep Human NK Cell Enrichment kit (StemCell Technologies). Purified peripheral blood NK cells (PBNK) and NK-92MI cells were plated at 10^6^ cells/ml in the NK-92MI culture medium; for peripheral NK cells, the medium was additionally supplemented with 20 ng/ml IL-2 and 10 ng/ml IL-15 (PeproTech). Cells were treated with either 2 μM 2-deoxy-D-glucose (2-DG; Sigma-Aldrich) or 40 nM oligomycin (Sigma-Aldrich). The use of the donor samples was approved by the Institutional Review Board of National Yang Ming Chiao Tung University (YM109023E) and was conducted in accordance with the Declaration of Helsinki.

### CD107a degranulation assay

NK cells were labeled with anti-CD56-FITC (clone HCD56, BioLegend 318,304) and anti-Siglec-7-APC antibodies (clone 6–434, BioLegend 339,206). Labeled NK cells were co-incubated with an equal number of K-562 target cells at 1:1 effector-to-target ratio at 37 °C for 1 h. After incubation, the cell mixture was stained with anti-CD107a-PE (clone eBioH4A3, Invitrogen 12–1079042). Samples were acquired on a CytoFLEX flow cytometer analyzed using CytExpert software (Beckman Coulter). CD107a expression was quantified in bulk, Siglec-7^NEG^, and Siglec-7^POS^ NK cell subsets, and data were normalized to the control condition.

### Mitochondrial mass measurement

Mitochondrial mass was assessed by incubating NK cells with 50 nM of MitoTracker Green FM (Thermo Fisher Scientific) for 30 min at 37 ˚C in a 5% CO_2_ incubator. Live samples were immediately acquired on CytoFLEX flow cytometer.

### Mitochondrial membrane potential measurement

Mitochondrial membrane potential (ΔΨm) was assessed using Tetramethylrhodamine ethyl ester (TMRE)-Mitochondrial Membrane Potential Assay Kit (Abcam) according to the manufacturer’s instructions. Cells were washed with PBS before staining, and cells treated with 50 μM carbonylcyanide-4-(trifluoromethyoxy) phenylhydrazone (FCCP) for 15 min were included as a depolarization positive control. Cells were then incubated with 150 nM TMRE for 30 min at 37 °C, after which the TMRE-containing medium was removed by centrifugation, and cells were washed with warm PBS. Cell pellets were resuspended in PBS, passed through a cell strainer, and acquired on a CytoFLEX flow cytometer. Data were analyzed using CytExpert and FlowJo software (BD Biosciences). Unstained samples were used to determine background fluorescence, which was used to normalize TMRE signals.

To assess the kinetics of change in ΔΨm upon Siglec-7 engagement, 5 × 10^4^ NK-92MI-S cells were seeded per well in 96-well plates and treated with QA79 (10 or 20 μg/ml; eBioQA79, Invitrogen 14–5759–37) or isotype control IgG1 (BioLegend 400,102) for 0, five or 10 min on ice. After washing with warm PBS, cells were incubated with TMRE in culture medium for 30 min at 37 °C. Following incubation, the TMRE solution was removed and cells were washed again with warm PBS. TMRE fluorescence was recorded on a SPARK multimode microplate reader (TECAN, Mannedorf) with 549 nm excitation and 575 nm emission. Background fluorescence from unstained control was subtracted from all measurements.

To assess the potential effect of sodium azide present in commercial antibody preparation, NK-92MI cells were incubated for 1 h at 37 °C with vehicle or sodium azide at 276, 552, 1380 μM. These concentrations correspond to the sodium azide levels present in 10, 20, or 50 μg/ml antibody preparations, respectively, assuming a 0.09% sodium azide stock solution dilutes 50-, 25-, or 10-fold. The 276 and 552 μM sodium azide concentrations correspond to those present in the 10 and 20 μg/ml QA79 preparations used in the functional assays, respectively, whereas 1380 μM sodium azide represents a higher control concentration corresponding to a 50 μg/ml antibody preparation. After treatment, cells were stained with MitoTracker Green FM and TMRE, followed by flow cytometric analysis.

### Protein extraction and immunoblotting

Cell lysates were prepared using lysis buffer (150 mM NaCl, 50 mM Tris, 1 mM EDTA, 1% Triton X-100) supplemented with protease/phosphatase inhibitor cocktail (Thermo Fisher Scientific) and incubated on ice for 20 min. Primary antibodies included anti-Drp1 (clone D6C7, Cell Signaling Technology 8570), anti-phospho-Drp1 (Ser^616^) (clone D9A1, Cell Signaling Technology 4494), anti-phospho-Drp1 (Ser^637^) (clone D3A4, Cell Signaling Technology 6319), anti-MFN1 (clone D6E2S, Cell Signaling Technology 14,739), anti-MFN2 (clone D1E9, Cell Signaling Technology 11,925), anti-ATG5 (clone EPR1755, Abcam ab108327), anti-LC3B (pAb, Abcam ab51520), anti-p62 (clone EPR4844, Abcam ab109012), anti-PARKIN (clone PRK8, GeneTex GTX39745) and anti-GAPDH (clone GA1R, Thermo Fisher Scientific MA5-15738). Bound antibodies were detected with horseradish peroxidase-conjugated anti-rabbit or anti-mouse IgG secondary antibodies (Cell Signaling Technology) and visualized using Western Lighting Chemiluminescence Reagent Plus (PerkinElmer).

### Flow cytometry

For surface receptor analysis, NK cells were stained with fluorophore-conjugated anti-CD56 (clone HCD56, BioLegend 318,304) or anti-Siglec-7 (clone 6–434, BioLegend 339,206) and acquired on a CytoFLEX flow cytometer.

For intracellular staining, cells were fixed with paraformaldehyde and stained with primary antibodies against phospho-Drp1 (Ser^616^) (clone D9A1, Cell Signaling Technology 4494) and phospho-Drp1 (Ser^637^) (clone D3A4, Cell Signaling Technology 6319), followed by Alexa Fluor 488-conjugated anti-rabbit IgG secondary antibody (Abcam ab150077).

For proliferation assay, PBNK cells were labeled with 1 μM carboxyfluorescein succinimidyl ester (CFSE; Molecular Probes) in PBS for 10 min at room temperature. The reaction was quenched by adding an equal volume of FBS, and cells were washed four times with complete medium (defined as day 0). After 7 days of culture with IL-15, CFSE-labeled NK cells were stained with anti-Siglec-7 and CFSE dilutions were assessed by flow cytometry.

For apoptosis analysis, cells were resuspended in Annexin V Binding Buffer (BD Biosciences), stained with Annexin V-PE and 7-AAD, and incubated for 15 min at 4 °C in the dark before flow cytometry analysis.

For detection of autophagic vacuoles, cells were stained using the CYTO-ID autophagy Detection Kit (Enzo Life Sciences, Farmingdale, NY, USA) according to the manufacturer’s instructions. Briefly, NK cells were washed once with assay buffer provided in the kit and resuspended at 1 × 10^6^ cells/ml. Anti-Siglec-7-APC (clone 6–434, BioLegend 339,206) and CYTO-ID Green detection reagent were added at a final dilution of 1:2000 and cells were incubated for 30 min at 37 °C in the dark. After incubation, cells were washed with assay buffer and analyzed on a CytoFLEX flow cytometer, and autophagic cells were identified based on increased CYTO-ID fluorescence intensity relative to unstained controls.

### Immunofluorescence staining and confocal microscopy

NK cells were seeded onto Cell-TAK (Corning)-coated glass-bottom dishes (Cellvis) and incubated for 45 min at 37 °C in the dark in prewarmed FACS buffer (2% FBS in PBS) containing 1.25 μM Hoechst 33,342, 400 nM MitoTracker Deep Red FM (Thermo Fisher Scientific), and 5 μg/ml anti-Siglec-7-PE (clone eBioQA79, Invitrogen 12–5759–42). After a single wash with prewarmed FACS buffer, cells were imaged using a Zeiss LSM880 laser-scanning confocal microscope operated in AiryScan mode.

Microscopy images were analyzed by ImageJ/Fiji software. Mitochondrial structures were segmented using the 2D threshold function of the Mitochondria Analyzer plugin, and the following parameters were quantified: mitochondrial number, mean area, mean form factor, mean aspect ratio, mean perimeter, mean branch length, branch junction and branch end point.

### Glycolysis and mitochondrial respiration assay

Glycolytic activity and mitochondrial respiration were measured using the Seahorse XF Glycolysis Stress Test Kit and Seahorse XF Cell Mito Stress Test Kit, respectively, on a Seahorse XF24 Analyzer (Agilent Technologies) according to the manufacturer's instructions. Briefly, 14 to 16 h before each assay, an XFe24 sensor cartridge was hydrated at 37 °C in a non-CO_2_ incubator. On the day of the assay, 24-well XF cell culture plates were coated with Cell-TAK for 20 min, washed, and 10^5^ NK cells were seeded per well.

Extracellular acidification rate (ECAR) was measured in XF medium under basal conditions and following sequential injections of 10 mM glucose, 10 mM oligomycin, and 50 mM 2-DG (all from Sigma-Aldrich). Glycolysis was defined as the difference between ECAR after glucose injection and ECAR after 2-DG injection; maximal glycolytic capacity was defined as the difference between ECAR after oligomycin and ECAR after 2-DG.

Oxygen consumption rate (OCR) was measured in XF assay medium (non-buffered DMEM containing 10 mM glucose, 2 mM L-glutamine, and 1 mM sodium pyruvate) under basal conditions and following sequential injections of 1 μM oligomycin, 1 μM FCCP, and 0.5 μM rotenone plus 0.5 μM antimycin A (Sigma-Aldrich). Basal respiration was defined as the difference between basal OCR and OCR after rotenone plus antimycin A; maximal respiration was defined as the difference between OCR after FCCP and OCR after rotenone plus antimycin A. Each measurement cycle consisted of 3 min mixing, 2 min waiting, and 3 min measurement. In selected experiments, isotype control antibody (BioLegend 400,102) or QA79 (clone eBioQA79, Invitrogen 14–5759–37) was added prior to the sequential injections.

### Statistical analysis and data representation

GraphPad Prism 10 software was used for graphical representation and statistical analysis. For comparison between two groups, paired and unpaired Student’s *t* test was used as appropriate. For comparison among more than two groups, one-way ANOVA followed by the appropriate multiple-comparison test was used. Data are presented as mean ± SD, and *p* < 0.05 was considered as statistically significant. Statistical significance is indicated as follows: ∗*p* < 0.05, ∗∗*p* < 0.01, ∗∗∗*p* < 0.001, ∗∗∗∗*p* < 0.0001.

## Data availability

All data generated or analyzed during this study are included in this published article and its supplementary information files.

## Supporting information

This article contains supporting information.

## Conflict of interest

The authors declare that they have no conflicts of interest with the contents of this article.

## References

[1] Lanier L.L. (2008). Up on the tightrope: natural killer cell activation and inhibition. Nat. Immunol..

[2] Pegram H.J., Andrews D.M., Smyth M.J., Darcy P.K., Kershaw M.H. (2011). Activating and inhibitory receptors of natural killer cells. Immunol. Cell Biol..

[3] Gardiner C.M., Finlay D.K. (2017). What fuels natural killers? Metabolism and NK cell responses. Front Immunol..

[4] Zhou L., Lin Q., Sonnenberg G.F. (2022). Metabolic control of innate lymphoid cells in health and disease. Nat. Metab..

[5] Detmer S.A., Chan D.C. (2007). Functions and dysfunctions of mitochondrial dynamics. Nat. Rev. Mol. Cell Biol.

[6] Buck M.D., O'Sullivan D., Pearce E.L. (2015). T cell metabolism drives immunity. J. Exp. Med..

[7] Fenton A.R., Jongens T.A., Holzbaur E.L.F. (2021). Mitochondrial dynamics: shaping and remodeling an organelle network. Curr. Opin. Cell Biol..

[8] Lin X.H., Qiu B.Q., Ma M., Zhang R., Hsu S.J., Liu H.H. (2020). Suppressing DRP1-mediated mitochondrial fission and mitophagy increases mitochondrial apoptosis of hepatocellular carcinoma cells in the setting of hypoxia. Oncogenesis.

[9] Moreno-Cubero E., Alrubayyi A., Balint S., Ogbe A., Gill U.S., Matthews R. (2024). IL-15 reprogramming compensates for NK cell mitochondrial dysfunction in HIV-1 infection. JCI Insight.

[10] Osuna-Espinoza K.Y., Rosas-Taraco A.G. (2023). Metabolism of NK cells during viral infections. Front Immunol..

[11] Terren I., Orrantia A., Astarloa-Pando G., Amarilla-Irusta A., Zenarruzabeitia O., Borrego F. (2022). Cytokine-induced memory-like NK cells: from the basics to clinical applications. Front Immunol..

[12] Li L., Mohanty V., Dou J., Huang Y., Banerjee P.P., Miao Q. (2023). Loss of metabolic fitness drives tumor resistance after CAR-NK cell therapy and can be overcome by cytokine engineering. Sci. Adv..

[13] Arellano-Ballestero H., Zubiak A., Dally C., Orchard K., Alrubayyi A., Charalambous X. (2024). Proteomic and phenotypic characteristics of memory-like natural killer cells for cancer immunotherapy. J. Immunother. Cancer.

[14] Kalia V., Yuzefpolskiy Y., Vegaraju A., Xiao H., Baumann F., Jatav S. (2021). Metabolic regulation by PD-1 signaling promotes long-lived quiescent CD8 T cell memory in mice. Sci. Transl Med..

[15] Patsoukis N., Bardhan K., Chatterjee P., Sari D., Liu B., Bell L.N. (2015). PD-1 alters T-cell metabolic reprogramming by inhibiting glycolysis and promoting lipolysis and fatty acid oxidation. Nat. Commun..

[16] Falco M., Biassoni R., Bottino C., Vitale M., Sivori S., Augugliaro R. (1999). Identification and molecular cloning of p75/AIRM1, a novel member of the sialoadhesin family that functions as an inhibitory receptor in human natural killer cells. J. Exp. Med..

[17] Ibarlucea-Benitez I., Weitzenfeld P., Smith P., Ravetch J.V. (2021). Siglecs-7/9 function as inhibitory immune checkpoints in vivo and can be targeted to enhance therapeutic antitumor immunity. Proc. Natl. Acad. Sci. U. S. A..

[18] Alphey M.S., Attrill H., Crocker P.R., van Aalten D.M. (2003). High resolution crystal structures of Siglec-7. Insights into ligand specificity in the Siglec family. J. Biol. Chem..

[19] Yamaji T., Mitsuki M., Teranishi T., Hashimoto Y. (2005). Characterization of inhibitory signaling motifs of the natural killer cell receptor Siglec-7: attenuated recruitment of phosphatases by the receptor is attributed to two amino acids in the motifs. Glycobiology.

[20] Shao J.Y., Yin W.W., Zhang Q.F., Liu Q., Peng M.L., Hu H.D. (2016). Siglec-7 defines a highly functional natural killer cell subset and inhibits cell-mediated activities. Scand. J. Immunol..

[21] Brunetta E., Fogli M., Varchetta S., Bozzo L., Hudspeth K.L., Marcenaro E. (2009). The decreased expression of Siglec-7 represents an early marker of dysfunctional natural killer-cell subsets associated with high levels of HIV-1 viremia. Blood.

[22] Rosenstock P., Horstkorte R., Gnanapragassam V.S., Harth J., Kielstein H. (2017). Siglec-7 expression is reduced on a natural killer (NK) cell subset of obese humans. Immunol. Res..

[23] Rodriguez E., Boelaars K., Brown K., Eveline Li R.J., Kruijssen L., Bruijns S.C.M. (2021). Sialic acids in pancreatic cancer cells drive tumour-associated macrophage differentiation via the Siglec receptors Siglec-7 and Siglec-9. Nat. Commun..

[24] Haas Q., Markov N., Muerner L., Rubino V., Benjak A., Haubitz M. (2022). Siglec-7 represents a glyco-immune checkpoint for non-exhausted effector memory CD8+ T cells with high functional and metabolic capacities. Front Immunol..

[25] Nguyen K.A., Hamzeh-Cognasse H., Palle S., Anselme-Bertrand I., Arthaud C.A., Chavarin P. (2014). Role of Siglec-7 in apoptosis in human platelets. PLoS One.

[26] Lin S.M., Wu S., Yu L.C., Twu Y.C. (2026). Siglec-7 links mitochondrial dynamics to megakaryocytic differentiation. Stem Cells. Dev..

[27] Mah A.Y., Rashidi A., Keppel M.P., Saucier N., Moore E.K., Alinger J.B. (2017). Glycolytic requirement for NK cell cytotoxicity and cytomegalovirus control. JCI Insight.

[28] Huang C.H., Liao Y.J., Fan T.H., Chiou T.J., Lin Y.H., Twu Y.C. (2018). A developed NK-92MI cell line with Siglec-7(neg) phenotype exhibits high and sustainable cytotoxicity against leukemia cells. Int. J. Mol. Sci..

[29] Wang Z., Guan D., Wang S., Chai L.Y.A., Xu S., Lam K.P. (2020). Glycolysis and oxidative phosphorylation play critical roles in natural killer cell receptor-mediated natural killer cell functions. Front Immunol..

[30] Highton A.J., Diercks B.P., Mockl F., Martrus G., Sauter J., Schmidt A.H. (2020). High metabolic function and resilience of NKG2A-Educated NK cells. Front Immunol..

[31] Hong S., Yu C., Rodrigues E., Shi Y., Chen H., Wang P. (2021). Modulation of Siglec-7 signaling via in situ-created high-affinity cis-Ligands. ACS Cent. Sci..

[32] Jandus C., Boligan K.F., Chijioke O., Liu H., Dahlhaus M., Demoulins T. (2014). Interactions between Siglec-7/9 receptors and ligands influence NK cell-dependent tumor immunosurveillance. J. Clin. Invest..

[33] Yoshimura A., Asahina Y., Chang L.Y., Angata T., Tanaka H., Kitajima K. (2021). Identification and functional characterization of a Siglec-7 counter-receptor on K562 cells. J. Biol. Chem..

[34] Nandagopal N., Ali A.K., Komal A.K., Lee S.H. (2014). The critical role of IL-15-PI3K-mTOR pathway in natural killer cell effector functions. Front Immunol..

[35] Mao Y., van Hoef V., Zhang X., Wennerberg E., Lorent J., Witt K. (2016). IL-15 activates mTOR and primes stress-activated gene expression leading to prolonged antitumor capacity of NK cells. Blood.

[36] Lin J.X., Du N., Li P., Kazemian M., Gebregiorgis T., Spolski R. (2017). Critical functions for STAT5 tetramers in the maturation and survival of natural killer cells. Nat. Commun..

[37] Cong J. (2020). Metabolism of natural killer cells and other innate lymphoid cells. Front Immunol..

[38] Surace L., Doisne J.M., Escoll P., Marie S., Dardalhon V., Croft C. (2021). Polarized mitochondria as guardians of NK cell fitness. Blood Adv..

[39] Preechanukul A., Saiprom N., Rochaikun K., Moonmueangsan B., Phunpang R., Ottiwet O. (2024). Metabolic requirements of CD160 expressing memory-like NK cells in Gram-negative bacterial infection. Clin. Transl Immunol..

[40] Zaghi E., Calvi M., Puccio S., Spata G., Terzoli S., Peano C. (2021). Single-cell profiling identifies impaired adaptive NK cells expanded after HCMV reactivation in haploidentical HSCT. JCI Insight.

[41] Huang Y.F., Su S.C., Chuang H.Y., Chen H.H., Twu Y.C. (2023). Histone deacetylation-regulated cell surface Siglec-7 expression promoted megakaryocytic maturation and enhanced platelet-like particle release. J. Thromb. Haemost..

[42] Dharmadhikari G., Stolz K., Hauke M., Morgan N.G., Varki A., de Koning E. (2017). Siglec-7 restores beta-cell function and survival and reduces inflammation in pancreatic islets from patients with diabetes. Sci. Rep..

