# Supplementary material for: Siglec-7 orchestrates mitochondrial dynamics and metabolic reprogramming to restrain human NK cell cytotoxic function
Source: J Biol Chem. 2026 Sep 8;302(10):113543. doi: 10.1016/j.jbc.2026.113543 (PMC13656541; doi:10.1016/j.jbc.2026.113543)
Supplement: Supplementary file 1 [file mmc1.docx]

**Supplemental data**

**Fig. S1 Metabolic inhibition of degranulation in Siglec-7^NEG^ and Siglec-7^POS^ NK cells.**

Peripheral NK cells (A) and NK-92MI cells (B) were gated into Siglec-7^NEG^ and Siglec-7^POS^ subsets and assessed for CD107a degranulation under control (CTR), 2-DG, or oligomycin (Oligo) treatment. Fractional inhibition was calculated for each donor and subset as (1- treated/CTR)$\times100$, and paired dot plots show inhibition (%) in Siglec-7^NEG^ and Siglec-7^POS^ cells [n=6 for (A), n=3 for (B)]. Statistical significance was determined by paired t-test; **P<0.05*; ***P<0.01*, ****P<0.001*.

**Fig. S2 Siglec-7 expression profiles in NK-92MI and NK-92MI-S cell lines.**

Flow cytometry dot plots showing the surface Siglec-7 expression in parental NK-92MI (15.2%) and NK-92MI-S (89.1%). The gated population indicates the percentage of Siglec-7^POS^ cells within each line. Data are represented one of 3 independent experiments.

**Fig. S3 Effect of sodium azide on mitochondrial mass and membrane potential.**

NK-92MI cells were treated with vehicle (CTR) or sodium azide at 276, 552, 1380 μM corresponding to the sodium azide concentrations present in 10, 20, or 50 μg/ml antibody preparations, respectively. The 276 and 552 μM concentrations correspond to the QA79 antibody used in the functional assays. Cells were then stained with MitoTracker Green FM (A) to assess mitochondrial mass and TMRE (B) to assess mitochondrial membrane potential, followed by flow cytometric analysis. Bar graphs show mean fluorescence intensity (MFI) for each condition, with individual points representing independent experiments (n=3); data represented as mean $\pm$ SD. Statistical comparison were performed using one-way ANOVA followed by Tukey’s multiple-comparison test.

**Fig. S4 Mitochondrial morphology of Siglec-7^NEG^ and Siglec-7^POS^ PBNK with QA79 engagement.**

PBNK were treated for 1 h with isotype control IgG1 or agonistic anti-Siglec-7 antibody (QA79), stained with Hoechst 33342, MitoTracker Green FM, and anti-Siglec-7, and imaged by confocal microscopy. Mitochondrial structures were segmented using the Mitochondria Analyzer plugin in ImageJ, and the indicated parameters were quantified in Siglec-7^NEG^ and Siglec-7^POS^ cells under each antibody condition. Data points represent individual cells polled from 10 independent donors; bars indicate mean ± SD. Statistical comparisons were performed using one-way ANOVA followed by Tukey’s multiple-comparison test; **P<0.05*; ***P<0.01*, ****P<0.001*, *****P<0.0001*.

**Fig. S5 Oxygen consumption rate (OCR) in Siglec-7^low^ and Siglec-7^high^ NK-92MI with QA79 engagement.**

(A) NK-92MI (Siglec-7^low^) and NK-92MI-S (Siglec-7^high^) cells were seeded on Cell-TAK-coated Seahorse XF24 plates and assayed using the Cell Mito Stress Test protocol. Cells were pretreated with isotype control IgG1 or QA79 for 1 h, and OCR was measured under basal conditions followed by sequential injections of oligomycin, FCCP, and R&A. Curves show mean OCR $\pm$ SD for IgG1-treated NK-92MI (blue), QA79-treated NK-92MI (dark blue), IgG1-treated NK-92MI-S (red), and QA79-treated NK-92MI-S (green) from three independent experiments.

(B) Basal respiration, maximal respiration, ATP production, spare respiration capacity, and proton leak were calculated per condition from the indicated phase of the Mito Stress test and are presented as bar graphs (mean $\pm$ SD). Statistical comparisons were performed using one-way ANOVA followed by Tukey’s multiple-comparison test; **P<0.05*; ***P<0.01*, ****P<0.001*.

**Fig. S6 QA79 selectively induces mitochondrial depolarization in Siglec-7^POS^ NK cells.**

NK-92MI cells were treated for 10 min with isotype control (IgG1) or agonistic anti-Siglec-7 antibody (QA79), followed by stained with anti-Siglec-7 (6-434) and TMRE to assess mitochondrial membrane potential. TMRE fluorescence histograms are shown for bulk IgG1-treated cells (IgG1 CTR, red), IgG1-treated Siglec-7^NEG^ (blue) and Siglec-7^POS^ (dark blue) gated subsets, bulk QA79-treated cells (QA79 CTR, green), QA79-treated Siglec-7^NEG^ (light purple) and Siglec-7^POS^ (purple) gated subsets. Numbers indicate mean TMRE fluorescence for each population. Data are represented one of 3 independent experiments.

**Fig. S7 Gating strategy for CYTO-ID staining in human NK cells.**

(A) Representative flow cytometry plots from one donor showing the gating strategy for PBNK isolation and Siglec-7 expression. PBNK was identified as CD3^-^CD56^+^ lymphocytes (left), and Siglec-7 expression was analyzed within gated NK cells using anti-Siglec-7 staining to define Siglec-7^NEG^ and Siglec-7^POS^ subsets (right). A representative histogram comparing NK cells stained with anti-Siglec-7 (black) and an isotype-matched control antibody (gray) is shown to illustrate the threshold used to define Siglec-7^NEG^ and Siglec-7^POS^ subsets.

(B) Flow cytometric analysis of CYTO-ID staining in NK cells. A control sample (CTR, unstained CD3^-^CD56^+^ NK cells) was used to define background autofluorescence in the CYTO-ID channel. In CYTO-ID-stained samples, CD3^-^CD56^+^ NK cells and Siglec-7^NEG^ and Siglec-7^POS^ subsets were analyzed by plotting CYTO-ID fluorescence (X-axis) versus side scatter (Y-axis). Autophagic cells were defined as CYTO-ID^hi^ cells based on increased fluorescence intensity along the X-axis, while SSC was used only to illustrate changes in cell granularity.

(C) Overlay histograms showing CYTO-ID fluorescence was collected in the FITC channel and displayed as relative fluorescence intensity.

**Fig. S8 CFSE-based proliferation analysis of Siglec-7^NEG^ and Siglec-7^POS^ subsets**

(A) Representative CFSE dilution histograms of bulk NK cells at day 0 and Siglec-7^NEG^ and Siglec-7^POS^ subset at day 7 under IL-15 stimulation are shown. CFSE intensity decreases with each round of cell division, allowing modeling of generation peaks using FlowJo.

(B) Summary table of proliferation metrics for Siglec-7^NEG^ and Siglec-7^POS^ populations cultured in IL-2 plus IL-15 and IL-15 alone. Metrics were obtained from FlowJo Proliferation platform and correspond to the CFSE dilution profiles shown in panel (A) and the main text (Fig. 7D). Data are representative of n=4 donors.

**Supplementary Table. Frequencies of Siglec-7^NEG^ and Siglec-7^POS^ NK cells in peripheral blood from healthy donors.**

Peripheral NK cells from healthy donors were analyzed by flow cytometry for surface CD3, CD56, and Siglec-7 expression. The table lists the percentages of Siglec-7^NEG^ and Siglec-7^POS^ NK cells within total NK cells for each donor (n=21).
